# Monitoring diaphragm activity and neuromechanical efficiency during acute respiratory failure: feasibility and preliminary findings

**DOI:** 10.1186/2197-425X-3-S1-A1002

**Published:** 2015-10-01

**Authors:** EC Goligher, C Urrea, S Vorona, LJ Brochard, C Sinderby, SS Bolz, GD Rubenfeld, BP Kavanagh, ND Ferguson

**Affiliations:** Division of Respirology, University Health Network, Toronto, Canada; Interdepartmental Division of Critical Care Medicine, University of Toronto, Toronto, Canada; Department of Physiology, University of Toronto, Toronto, Canada; Keenan Centre for Biomedical Research, St. Michaels Hospital, Toronto, Canada; Department of Critical Care, Sunnybrook Health Sciences Centre, Toronto, Canada; Department of Critical Care Medicine, Hospital for Sick Children, Toronto, Canada

## Introduction

Ventilator-induced diaphragm dysfunction is thought to result from diaphragm inactivity and/or dyssynchronous eccentric diaphragm contractions during mechanical ventilation [1, 2]. The magnitude of diaphragm disuse and the frequency of eccentric contractions and their impact on diaphragm function remains uncertain.

## Objectives

To describe the feasibility of monitoring diaphragm activity, patient-ventilator synchrony, and the efficiency of diaphragm pressure generation longitudinally during the first week of mechanical ventilation.

## Methods

Patients requiring invasive mechanical ventilation due to sepsis, ARDS, or severe acute brain injury were enrolled within 24 hours of intubation. Airway pressure, flow, crural diaphragm EMG activity (Edi) and transdiaphragmatic pressure (Pdi) were recorded for 5 minutes of every hour for up to 7 days. To assess for changes in diaphragm function, neuromechanical efficiency (NME - ratio of Pdi/Edi) was measured on a daily basis [3]. Diaphragm thickness (Tdi) and thickening fraction (TFdi) were measured once daily [4].

## Results

Ten patients (6 female) were enrolled over 8 months: 3 were intubated for intracranial hemorrhage, 5 for pneumonia, and 2 for non-pulmonary sepsis. Five subjects completed 7 days of monitoring; 2 were successfully extubated and 3 died within 7 days (total patient-days: 52). Edi was initially very low (< 5 uV) or absent in 6 of 10 subjects and tended to increase over several days. Daily TFdi was strongly correlated with the 24-hour average value of Edi (conditional R^2^ = 0.65, p = 0.04). NME could not be measured on 24 patient-days (46%) owing to the absence of Edi activity. Baseline NME varied between patients and NME decreased over time in some patients.

## Conclusions

It is feasible to monitor diaphragm activity, synchrony and pressure generating efficiency longitudinally during mechanical ventilation. However, many subjects did not complete 7 days of monitoring and NME could not be measured on almost 50% of study days owing to absent diaphragm activity. Changes in diaphragm thickening fraction measured once daily reflect variation in average daily diaphragm activity.

## Grant Acknowledgment

This study was supported by funding from the Physician Services Incorporated (PSI) Foundation.
